# Supplementary material for: Sexual reproduction of the snow alga Chloromonas fukushimae (Volvocales, Chlorophyceae) induced using cultured materials
Source: PLoS One. 2020 Aug 26;15(8):e0238265. doi: 10.1371/journal.pone.0238265 (PMC7449499; doi:10.1371/journal.pone.0238265)
Supplement: S1 Fig — (A) Brown snow in Mt. Hakkoda, Aomori, Japan (40°38’49.50" N, 140°51’04.70" E) on May 18, 2016. (B) Green snow in Oze National Park, Gunma, Japan (36°54’32.91" N, 139°11’50.81" E) on April 29, 2017. (DOCX) [file pone.0238265.s001.docx]

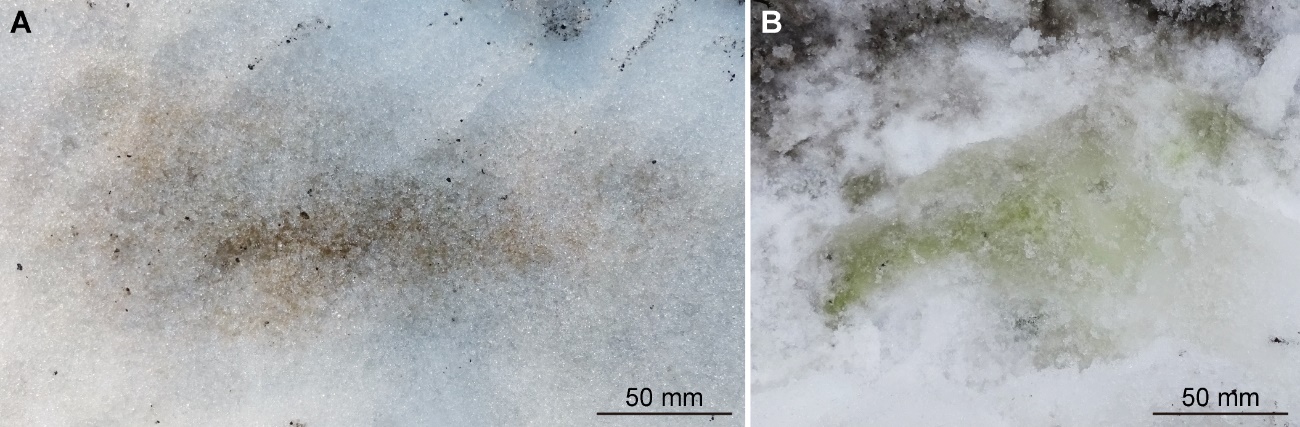


**S1 Fig. Colored snow materials examined in this study.** (A) Brown snow in Mt. Hakkoda, Aomori, Japan (40°38'49.50" N, 140°51'04.70" E) on May 18, 2016. (B) Green snow in Oze National Park, Gunma, Japan (36°54'32.91" N, 139°11'50.81" E) on April 29, 2017.
