# Supplementary material for: Sexual reproduction of the snow alga Chloromonas fukushimae (Volvocales, Chlorophyceae) induced using cultured materials
Source: PLoS One. 2020 Aug 26;15(8):e0238265. doi: 10.1371/journal.pone.0238265 (PMC7449499; doi:10.1371/journal.pone.0238265)
Supplement: S2 Fig — Serial phase contrast micrographs, with identical magnification throughout. Brightness and contrast were adjusted by Adobe Photoshop CS5 (Adobe Inc., California, USA) for showing entwined flagella (asterisks) and a cell wall which was being released from a gamete (arrowhead). Strains HkCl-117 (mt+) and NIES-3390 (mt−) were used. The times after beginning the observations were denoted at the upper right of each photograph. The mating types of gametes were not confirmed. (DOCX) [file pone.0238265.s002.docx]

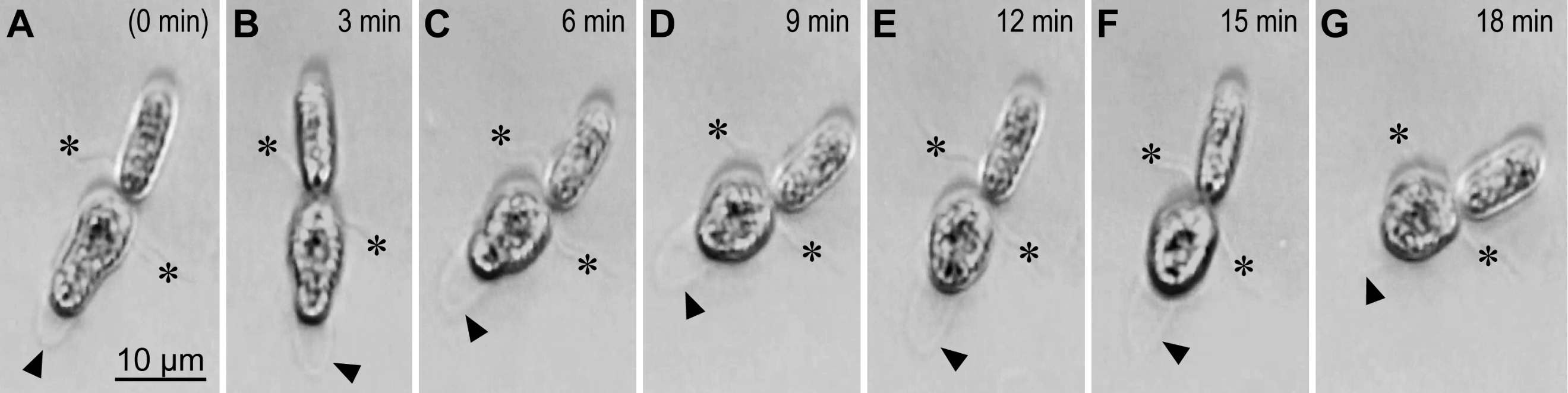


**S2 Fig. Morphological change in a gamete of the mating pair in *Chloromonas fukushimae*.** Serial phase contrast micrographs, with identical magnification throughout. Brightness and contrast were adjusted by Adobe Photoshop CS5 (Adobe Inc., California, USA) for showing entwined flagella (asterisks) and a cell wall which was being released from a gamete (arrowhead). Strains HkCl-117 (mt+) and NIES-3390 (mt−) were used. The times after beginning the observations were denoted at the upper right of each photograph. The mating types of gametes were not confirmed.
