# Supplementary material for: Sexual reproduction of the snow alga Chloromonas fukushimae (Volvocales, Chlorophyceae) induced using cultured materials
Source: PLoS One. 2020 Aug 26;15(8):e0238265. doi: 10.1371/journal.pone.0238265 (PMC7449499; doi:10.1371/journal.pone.0238265)
Supplement: S3 Fig — Differential interference contrast micrographs with identical magnification throughout. Strains UTEX SNO91 (mt+) and UTEX SNO92 (mt−) were used under nitrogen-sufficient condition (AF-6 medium). Cell fixation was performed, using 0.4% of Lugol’s iodine solution. An asterisk indicates entwined flagella. (A) The pairing of two elongate gametes (fixed). (B) The pairing of a spherical gamete and an elongate gamete (fixed). Note that the spherical gamete was smaller than the elongate one. (C) The pairing of a spherical gamete and an elongate gamete (fixed). Note that the spherical gamete was larger than the elongate one. (D) The pairing of two spherical gametes (fixed). (E) Quadriflagellate planozygote (fixed). Three of the four flagella are shown. (F) Mature aplanozygote, accumulating orange pigments within the cell. (DOCX) [file pone.0238265.s003.docx]

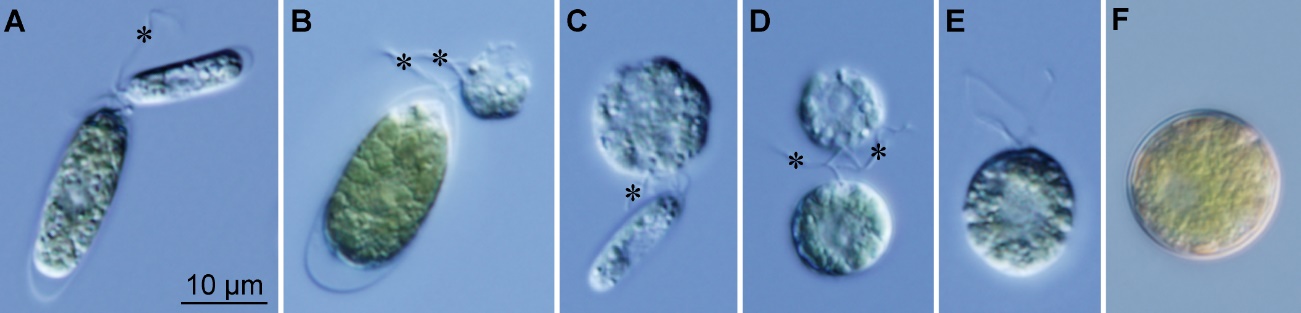


**S3 Fig. Sexual reproduction of *Chloromonas tughillensis*.** Differential interference contrast micrographs with identical magnification throughout. Strains UTEX SNO91 (mt+) and UTEX SNO92 (mt−) were used under nitrogen-sufficient condition (AF-6 medium). Cell fixation was performed, using 0.4% of Lugol’s iodine solution. An asterisk indicates entwined flagella. (A) The pairing of two elongate gametes (fixed). (B) The pairing of a spherical gamete and an elongate gamete (fixed). Note that the spherical gamete was smaller than the elongate one. (C) The pairing of a spherical gamete and an elongate gamete (fixed). Note that the spherical gamete was larger than the elongate one. (D) The pairing of two spherical gametes (fixed). (E) Quadriflagellate planozygote (fixed). Three of the four flagella are shown. (F) Mature aplanozygote, accumulating orange pigments within the cell.
