# Supplementary material for: Sexual reproduction of the snow alga Chloromonas fukushimae (Volvocales, Chlorophyceae) induced using cultured materials
Source: PLoS One. 2020 Aug 26;15(8):e0238265. doi: 10.1371/journal.pone.0238265 (PMC7449499; doi:10.1371/journal.pone.0238265)
Supplement: S1 Table — (DOCX) [file pone.0238265.s004.docx]

**S1 Table. Results of mating experiments among strains of *Chloromonas fukushimae*.**

|  | HkCl-106 | HkCl-108 | HkCl-113 | HkCl-117 | HkCl-121 | HkCl-125 | OzCl-11 | NIES-3389 | NIES-3390 |
| --- | --- | --- | --- | --- | --- | --- | --- | --- | --- |
| HkCl-106 | − | − | − | − | − | − | M | − | M |
| HkCl-108 |  | − | − | − | − | − | M | − | M |
| HkCl-113 |  |  | − | − | − | − | M | − | M |
| HkCl-117 |  |  |  | − | − | − | M | − | M |
| HkCl-121 |  |  |  |  | − | − | M | − | M |
| HkCl-125 |  |  |  |  |  | − | M | − | M |
| OzCl-11 |  |  |  |  |  |  | − | − | − |
| NIES-3389 |  |  |  |  |  |  |  | − | − |
| NIES-3390 |  |  |  |  |  |  |  |  | − |

For the method for induction of sexual reproduction, see Materials and Methods.

Explanation of symbols: M, mating of gametes was observed within a day; −, mating of gametes was not observed within 3 days.
